# Schwannoma of the Ascending Colon in a 22-Year-Old Male: A Case Report

**DOI:** 10.7759/cureus.31946

**Published:** 2022-11-27

**Authors:** Rimsha R Vohra, Syed Faqeer Hussain Bokhari, Mohammad Owais, Muhammad Haseeb, Fatimah Kharal

**Affiliations:** 1 Internal Medicine, Dow University of Health Sciences, Karachi, PAK; 2 Medicine and Surgery, Mayo Hospital, Lahore, PAK; 3 Medicine, Mayo Hospital, Lahore, PAK; 4 Medicine, Jinnah Hospital Lahore, Lahore, PAK; 5 Medicine, Bahria International Hospital, Lahore, PAK; 6 Internal Medicine, CMH (Combined Military Hospitals) Lahore Medical College and Institute of Dentistry, Lahore, PAK

**Keywords:** nerve sheath tumor, sox 10, positive s 100, neurilemmoma, neurofibromatosis ii, benign colonic polyp, colonoscopy, gastrointestinal schwannoma, ascending colon, schwannoma

## Abstract

Schwannoma of the ascending colon is an extremely rare neoplasm that is often discovered incidentally in the asymptomatic older population on surveillance colonoscopy. We present the case of a symptomatic 22-year-old male presenting with one month of discomfort in the right lower abdominal quadrant, abdominal bloating, and hematochezia. A sessile polyp measuring 0.5 cm was identified in the ascending colon on the colonoscopy. The polyp was completely resected using cold snare polypectomy. Histological examination of the resected polyp with hematoxylin-eosin staining revealed small nodules of bland spindle cells with focal nuclear condensation. The lesional cells tested positive for S-100 and SOX-10 on immunohistochemical analysis, establishing the diagnosis of benign schwannoma. Since this lesion was submucosal, its diagnosis required an endoscopic biopsy that could only be performed on the mucosa. It was difficult to distinguish it from other mesenchymal tumors (gastrointestinal stromal tumor or leiomyoma), and this makes the differential diagnosis exceedingly challenging. If the immunohistochemistry is positive for S-100 and negative for C-KIT, CD-34, actin, and desmin, it aids in diagnosis. These tumors have non-specific radiological features and are asymptomatic.

## Introduction

Schwannoma is a neoplasm arising from Schwann cells and can occur anywhere along the peripheral nerves in the body [1]. However, gastrointestinal schwannoma (GIS), particularly of the ascending colon, is extremely rare [2]. GIS originates from Schwann cells that form the neural sheath surrounding the myenteric plexuses. It is typically a benign and slow-growing mass, with minimal risk of malignancy [3,4]. This uncommon tumor makes up 2-6% of all mesenchymal tumors in the gastrointestinal tract [5,6]. Owing to the absence of any distinctive feature, GIS is difficult to diagnose radiologically or endoscopically [7,8]. Mucosal biopsies are insufficient to distinguish these from other gastrointestinal tumors. Immunohistochemical analysis is performed to establish a conclusive diagnosis [8]. Complete excision with wide margins is a mandatory intervention for GIS due to its tendency to recur locally or become malignant if left untreated [9]. This study reports a rare case of Schwannoma of the ascending colon in a 27-year-old male. It was detected on a colonoscopy and was completely excised. The diagnosis was established via immunohistopathological analysis of the excised polyp.

## Case presentation

**Figure 1:**
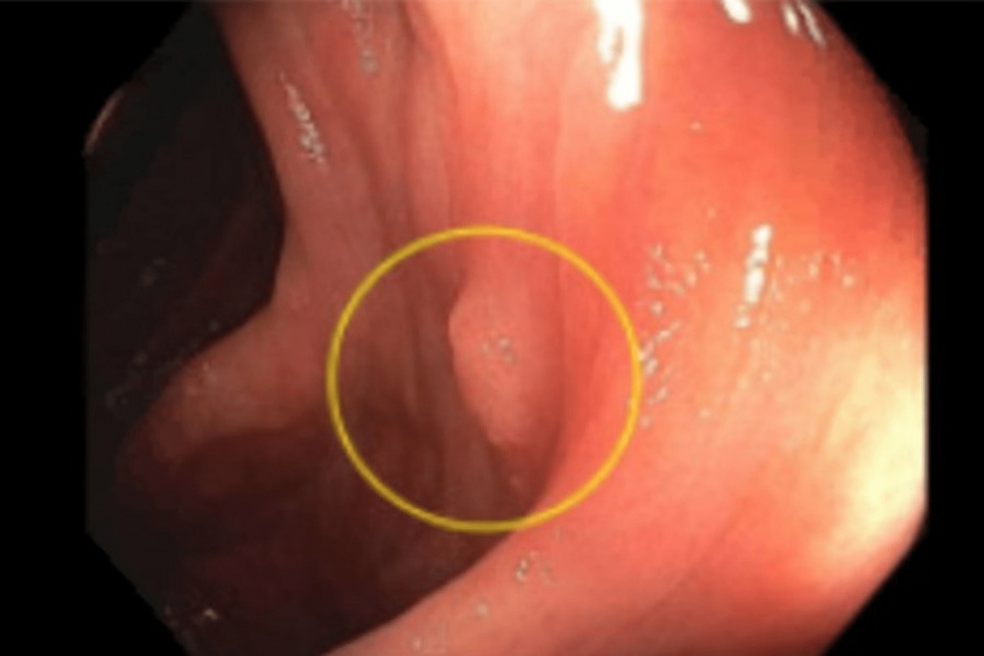
A sessile polyp observed on the ascending colon during colonoscopy.

A previously healthy 22-year-old male with a one-month history of abdominal bloating and mild discomfort in the right lower abdominal quadrant presented with hematochezia. Physical examination and laboratory evaluation showed no significant findings. Abdominal ultrasound revealed fatty liver and two gallstones, each measuring 3 mm. The family history of the patient was significant, with a diagnosis of colon cancer in the father (at 47 years of age) and celiac disease in the brother. Ultrasound and CT scan findings of the abdomen were normal. Esophagogastroduodenoscopy was also insignificant. Therefore, a colonoscopy was performed and revealed a sessile polyp measuring 0.5 cm on the ascending colon, approximately at a distance of 8.4 cm from the cecum (Figure 1). The polyp was considered to be the cause of hematochezia since no other significant findings were present. The differential diagnoses included lymphoma, leiomyoma, and adenoma.

**Figure 2:**
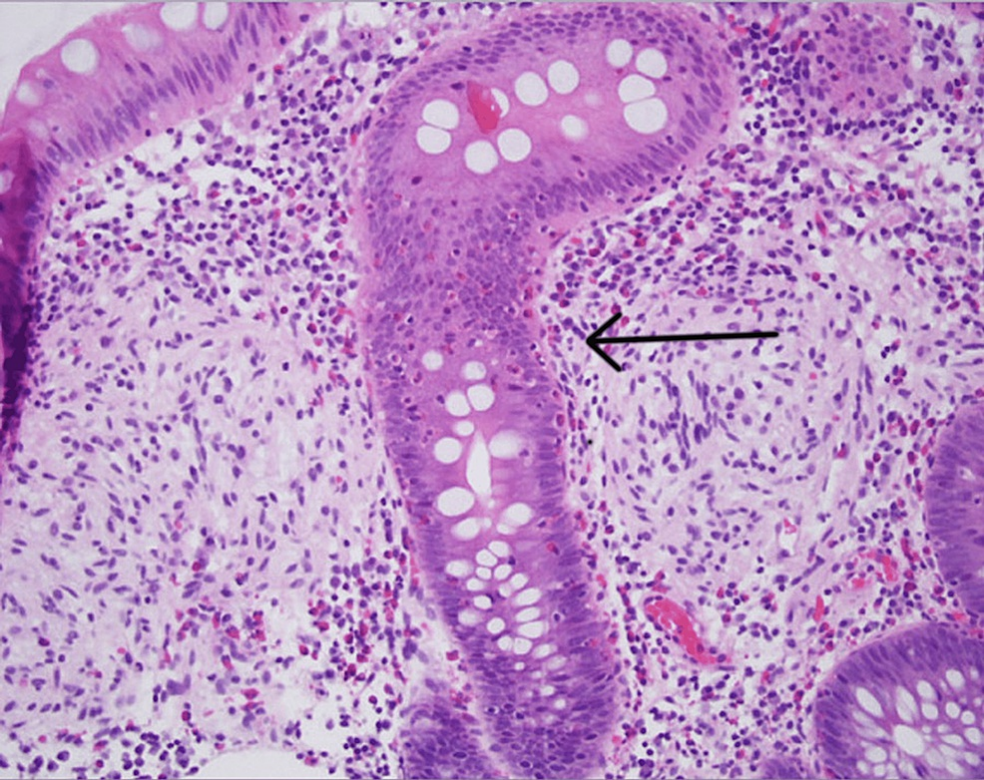
H&E stain showing small nodules of bland spindle cells with focal nuclear condensation (arrow pointing at the verocay body).

The polyp was removed completely with a cold snare polypectomy. Histopathological examination revealed small nodules of bland spindle cells with focal nuclear condensation suggestive of a verocay body (Figure 2).

**Figure 3:**
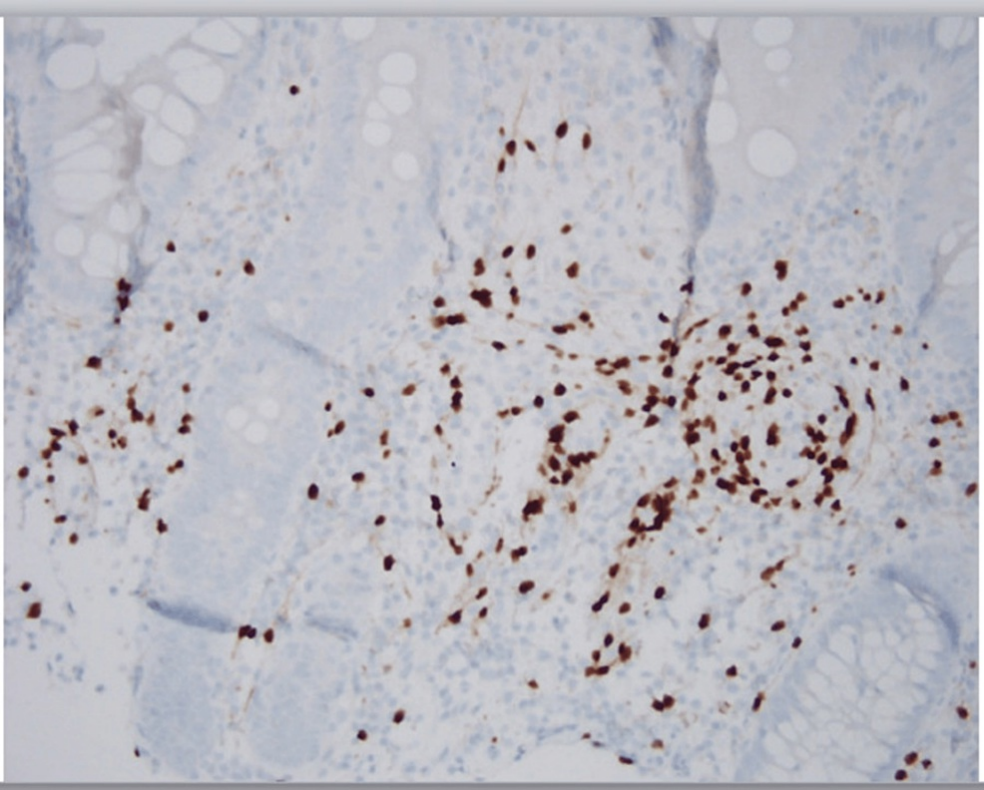
Lesional cells strongly positive for SOX-10 protein immunohistochemical stain (dark brown color).

**Figure 4:**
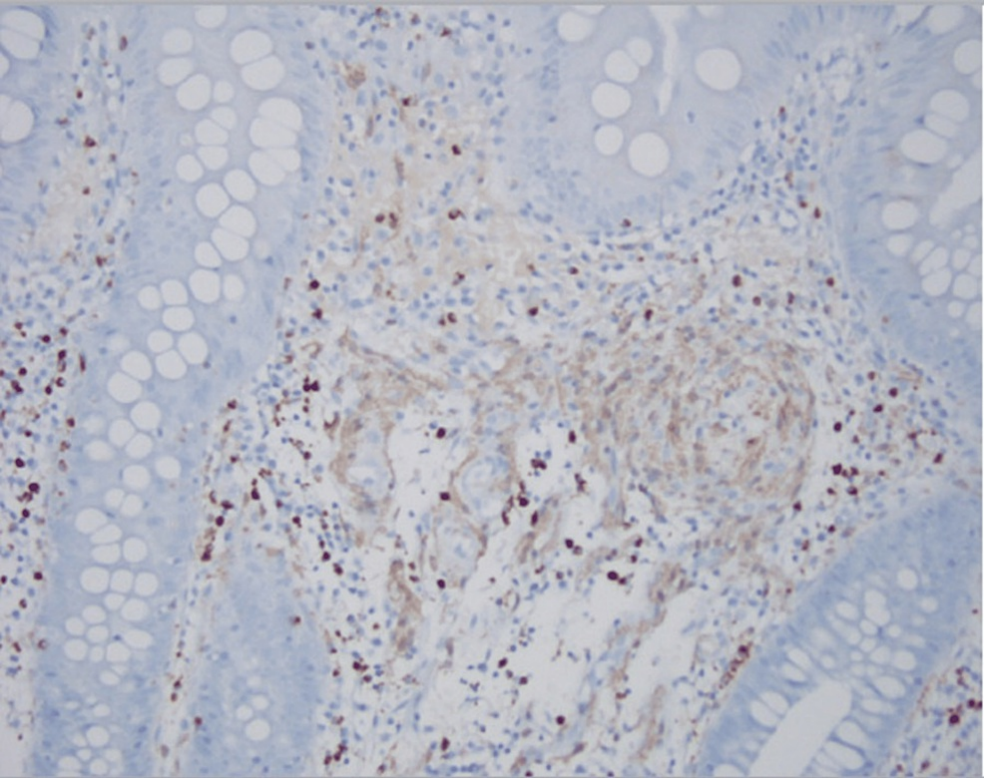
Lesional cells mildly positive for S-100 protein immunohistochemical stain (light brown color).

Immunohistochemical analysis revealed that the lesional cells were strongly positive for SOX-10 (Figure 3) and mildly positive for S-100 protein (Figure 4). No dysplasia or malignancy was observed. Based on these findings, a conclusive diagnosis of colonic schwannoma was established. The patient had a good prognosis without bowel obstruction and no signs of infection. An oral diet was initiated diet after two days. The patient was discharged with no complaints and was recommended to seek outpatient care. During the two-year follow-up, there was no recurrence.

## Discussion

Schwannomas are exceedingly rare neoplasms that arise from Schwann cells in the nerve sheath. Gastrointestinal schwannomas (GIS) appear as spindle cell tumors in the gastrointestinal system and originate from Auerbach’s myenteric plexus rather than Meissner’s submucosal plexus. They account for 2-6% of all mesenchymal cancers [10]. Even among GIS, schwannoma of the ascending colon is an extremely rare finding [10,11]. Most of them are benign and asymptomatic and are usually identified during screening colonoscopies or CT scans for other reasons. Depending on the size and location of the tumor, symptoms may include rectal bleeding, abdominal pain, constipation, and tenesmus [1].

Schwannoma rarely ulcerates into the mucosa and is frequently identified as a submucosal mass or polyp on a colonoscopy or CT scan [12]. Mucosal biopsy, like that of all other mesenchymal tumors, is frequently inconclusive. Deep biopsy or submucosal resection might help distinguish schwannoma from other mesenchymal cancers, such as gastrointestinal stromal tumors, neuroendocrine tumors, leiomyomas, and leiomyoma-sarcomas, or from adenocarcinomas in ulcerated mucosa [13]. Schwannomas can range in size from less than 1 cm lesions to bulky tumors of up to 28 cm in diameter that can cause an increase in abdominal girth [14]. A sessile polyp measuring 0.5 cm on the ascending colon was observed in this case.

Postsurgical immunohistopathologic evaluation of the excised polyp yields a conclusive diagnosis [8]. Schwannoma stains were positive for S-100 and SOX-10 but negative for SMA, desmin, CD117, and P53 [15,16]. This case showed an Antoni A-type histologic pattern, which was confirmed by the presence of verocay bodies during the H&E examination of excised mass, and was positive for S-100 and SOX-10 proteins [11].

Complete surgical excision with free negative margins is the best treatment option for colonic schwannoma [7]. Radical surgery is not required. The high prevalence of radical resection found in the literature is because of the lack of an appropriate preoperative diagnosis [7]. Schwannomas are removed either endoscopically or by a wedge resection when identified preoperatively [9,17]. In our case, we used cold snare polypectomy to excise the tumor endoscopically, which led to a good prognosis.

## Conclusions

Colonic schwannomas are rare, slow-growing benign neoplasms that usually occur in the older population. This case report showed that colonic schwannoma may also present in younger populations and has a very vague clinical presentation. It is usually identified on a colonoscopy or CT scan. Postsurgical immunohistopathologic examination of the excised mass is used to establish a conclusive diagnosis. Endoscopic excision or wedge resection is the treatment of choice.
